# Reply: Oxaliplatin- or irinotecan-based chemotherapy for metastatic colorectal cancer in the elderly

**DOI:** 10.1038/sj.bjc.6601807

**Published:** 2004-04-13

**Authors:** T Aparicio, E Mitry, J Ezenfis, S Dominguez

**Affiliations:** 1 Hôpital Bichat-Claude Bernard, AP-HP, Service d'Hepato-Gastroenterologie, 46 rue Henri Huchard, Paris 75018, France; 2 Service d' Hépeto-gastroentérologie, Hôpital Ambraise Peré, AP-HP, Boulogue 92100, France; 3 Service d' Hépeto-gastroentérologie, Hôpital ole Longjumean, Longjumean S1600, France; 4 Départment Uro-Digesvif, Centre Oscan Lambret, Lille S9020, France

**Sir**,

Dr Alliot and Barrios pointed out that our results have been obtained in selected elderly patients. We did not evaluate the number of elderly patients who were candidates for oxaliplatin or irinotecan chemotherapy but did not receive it. Nevertheless, two French studies estimate that only 8–17% of patients over 75 years with advanced colorectal cancer received palliative chemotherapy (Mitry *et al*, 2003; Navazesh *et al*, 2003). Few elderly patients are referred to the oncologist for various reasons (Mahoney *et al*, 2000). In our study, the patients have few comorbidities and we concluded that chemotherapy with oxaliplatin or irinotecan is feasible in fit elderly patients.

The overall rate of grade III and IV toxicity appears too high to be acceptable for Dr Alliot and Barrios. Nevertheless, only 17% of the patients stopped the treatment because of the toxicity and no toxic death occurred after the 545 cycles analysed. Moreover, the observed toxicity rate was similar to that observed in younger patients treated with these drugs in a palliative situation (de Gramont *et al*, 2000; Douillard *et al*, 2000). The toxicity of irinitocan is related to the administration schedule even in young patients. The two phase II studies cited by Alliot and Barrios (Rougier *et al*, 1997; Rothenberg *et al*, 1999) investigated weekly or 3-weekly irinotecan monotherapy schedule. The weekly regimen is more toxic than the biweekly schedule and dose reduction is less frequent in the latter (Douillard *et al*, 2000).

The mean haemoglobin level was in the lower bound of the normal range, creatinin clearance was reduced in our patients and especially in those aged over 80, as it is presented in Table 1. Bilirubin was increased in 9% of the patients and alkaline phosphatase increased more than 2 N in 15% of the patients. Nevertheless, neither haemoglobin level, creatinin clearance, alkaline phosphatase nor bilirubin level were associated with increased toxicity in our patients (data not shown). It must be pointed out that patients with a Charlson score >2 had more treatment interruption for toxicity.

We agree with Dr Alliot and Barrios that albumin level and polypharmacy may interfere with chemotherapy and that a dose reduction should be done in some cases. In our study, only six (9%) patients had a dose reduction from the first cycle, a severe toxicity occurred in only one (17%) of these patients. Early toxicity within the first two cycles of chemotherapy leading to a dose reduction occurred in eight (12%) patients. So, it appears that not all elderly patients will benefit from a systematic initial dose reduction. Nevertheless, the predictive factors for adaptation could not be drawn from this study.

A recent study demonstrated that the willingness of elderly patients to be treated with chemotherapy is high even in French patients and must not be underestimated (Extermann *et al*, 2003). The only way to improve the management and prognostics of elderly patients with cancer and to evaluate the safety and efficiency of chemotherapy in this population is to perform specific trials. Two prospective randomised studies specific for elderly patients with advanced colorectal cancer have just started in France, initiated by the ‘Fédération Francophone de Cancérologie Digestive’. So, we invite our French colleagues to join us and include their patients in these studies in order to precise the best therapeutic strategy for elderly patients.
